# Adipokines and the Female Reproductive Tract

**DOI:** 10.1155/2014/232454

**Published:** 2014-02-18

**Authors:** Maxime Reverchon, Christelle Ramé, Michael Bertoldo, Joëlle Dupont

**Affiliations:** ^1^INRA, UMR85 Physiologie de la Reproduction et des Comportements, 37380 Nouzilly, France; ^2^CNRS, UMR6175 Physiologie de la Reproduction et des Comportements, 37380 Nouzilly, France; ^3^Université François Rabelais de Tours, 37041 Tours, France; ^4^IFCE, 37380 Nouzilly, France

## Abstract

It is well known that adipose tissue can influence puberty, sexual maturation, and fertility in different species. Adipose tissue secretes molecules called adipokines which most likely have an endocrine effect on reproductive function. It has been revealed over the last few years that adipokines are functionally implicated at all levels of the reproductive axis including the gonad and hypothalamic-pituitary axis. Many studies have shown the presence and the role of the adipokines and their receptors in the female reproductive tract of different species. These adipokines regulate ovarian steroidogenesis, oocyte maturation, and embryo development. They are also present in the uterus and placenta where they could create a favorable environment for embryonic implantation and play a key role in maternal-fetal metabolism communication and gestation. Reproductive functions are strongly dependent on energy balance, and thereby metabolic abnormalities can lead to the development of some pathophysiologies such as polycystic ovary syndrome (PCOS). Adipokines could be a link between reproduction and energy metabolism and could partly explain some infertility related to obesity or PCOS.

## 1. Introduction

It is now recognized that the white adipose tissue is a multifunctional organ. In addition to its key role of lipid storage, it has a crucial endocrine function secreting many hormones called adipokines [1]. These molecules are cytokines produced in the main by adipocytes or adipose stromal cells. Adipokines are implicated in adipocyte differentiation, energy metabolism, insulin resistance, inflammation, immunity, cancer, and angiogenesis [2–5]. It is well known that an excess or deficiency of white adipose tissue affects puberty, sexual maturation, and fertility in different species [6]. Furthermore, variations of white adipose tissue quantities modulate the expression level and serum concentrations of adipokines.

Obesity and excess weight are significantly involved in the decline in the natural fertility of mammals. New roles of adipokines have recently emerged in the field of fertility and reproduction [7]. Indeed, adipokines such as leptin, adiponectin, and resistin are able to regulate the functions of gonads and the hypothalamic-pituitary axis [8, 9]. Furthermore, the reproductive tract is tightly coupled with energy balance, and thereby metabolic abnormalities can lead to the development of some pathophysiologies such as polycystic ovary syndrome (PCOS) [10]. PCOS is the commonest endocrine disorder in women, affecting 5–10% of females of reproductive age. In this review, we focus on the localization and the role of some adipokines (in particular, adiponectin, resistin, visfatin, and chemerin) and their receptors on the female reproductive tract including ovary, placenta, and uterus. Finally, we will discuss their potential as actors involved in PCOS.

### 1.1. Adiponectin

**Figure 1:**
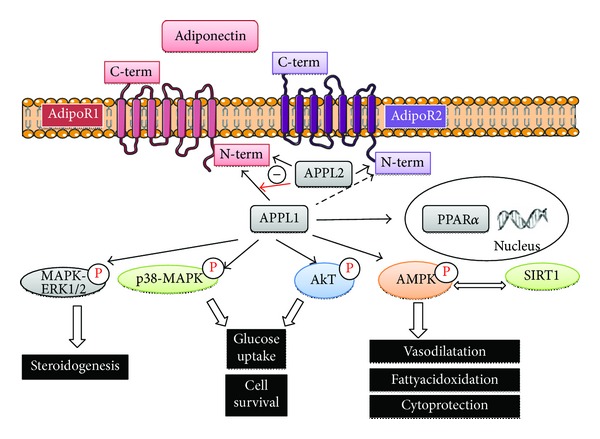
Signalling transduction via adiponectin receptors (AdipoR1 and AdipoR2) activation. The binding of the different forms of adiponectin to the two known adiponectin receptors, AdipoR1 and AdipoR2, can lead to stimulation of various signaling pathways. Indeed, interacting directly with the N-terminal of at least AdipoR1 and possibly AdipoR2, APPL1 elicits signalling through not only PPAR*α*, AMPK, and AMPK/SIRT1 but also p38-MAPK, ERK1/2-MAPK, and Akt. APPL2 binds to AdipoR1 and AdipoR2. Unlike APPL1, APPL2 inhibits AdipoR1 dependent signaling. According to the tissue, activation of both receptors results in modulation of different biological effects such as steroidogenesis, glucose uptake, cell survival, fatty acid oxidation, vasodilatation, and cytoprotection. APPL1/2: adaptor protein containing pleckstrin homology domain, phosphotyrosine binding domain, and leucine zipper motif 1; PPAR*α*: peroxisome proliferator-activated receptor *α*; SIRT1: sirtuin 1 (a NAD-dependent deacetylase); AMPK: 5′ adenosine monophosphate-activated protein kinase; MAPK: Mitogen-activated protein kinase; ERK1/2: extracellular signal-regulated kinases 1/2 (−: inhibition).

Adiponectin was discovered in 1995 after leptin. It is a protein of 244 amino acids (30 kDa) produced mainly by white adipose tissue but is also found in other tissues such as muscle and bone [11, 12]. Adiponectin is also known as Acpr30 (adipocyte complement-related protein 30 kDa), apM1 (adipose most abundant gene transcript-1), or GBP 28 (gelatin-binding protein) [13]. Although adiponectin is secreted mainly by the adipose tissue, more recent studies have indicated that it is more widely expressed in various species [14, 15]. In humans, the expression level of adiponectin mRNA varies depending on its location since expression is lower in visceral adipose tissue as opposed to subcutaneous adipose tissue [16]. Unlike other adipocyte-derived hormones, adiponectin gene expression and blood concentrations are inversely associated with body mass index [17]. In serum, adiponectin assembles into several oligomeric multimers including trimers, known as low molecular weight (LMW); hexamers, known as medium molecular weight (MMW) and higher molecular weight (HMW) multimeric complexes [18]. HMW are considered the most biologically active isoforms [13, 18]. Adiponectin structurally belongs to the complement 1q family and is found at high concentrations (>0.01% of the total protein) in serum of healthy individuals [13]. It is well known for its effect in improving insulin sensitivity [19] and regulating various processes including lipid synthesis, energy homeostasis, vasodilatation, and atherogenic activity [13, 19]. Adiponectin acts mainly through two G-coupled receptors named AdipoR1 and AdipoR2 (Figure 1). Interestingly the intracellular/extracellular orientation of the N-terminus and C-terminus is the opposite of classical G-coupled receptors. In addition, T-cadherin has also been reported to serve as a receptor for high-order multimers of adiponectin [20]. In HEK293 cells, transcriptional down regulation of T-cadherin largely improves adiponectin-mediated ERK1/2 activation suggesting that T-cadherin either competes with AdipoR1/R2 for adiponectin binding or interferes with the coupling of adiponectin-bound AdipoR1/R2 to downstream effectors [21]. However, a more detailed analysis of the adiponectin/T-cadherin function remains to be determined.

Downstream of AdipoR1 and AdipoR2: the biological effects of adiponectin are mediated by different signaling pathways involving the following molecules: AMPK, PPAR*γ* ERK, AKT, and P38 [13, 22]. Besides these signaling pathways, APPL1 and APPL2 [23], Ca2+, and SIRT1 [24] are emerging downstream effectors of the AdipoRs [25]. AdipoR signaling can be modulated by the interaction with two adaptor proteins: adaptor protein containing pleckstrin homology domain, phosphotyrosine binding domain, and leucine zipper motif 1 (APPL1 and APPL2) (Figure 1). Following adiponectin-AdipoR1 binding, APPL1 mediates a number of downstream signaling events associated with adiponectin function [26]. When the receptor is inactive APPL2 binds and inhibits APPL1 function, but APPL2 binding is displaced upon activation of AdipoR1 [27]. Thus, adiponectin, which is an abundant circulating protein synthesized mainly in adipose tissue, appears to be a major modulator of insulin action.

### 1.2. Resistin

Resistin was identified in 2001 by Steppan et al. [28]. It is a circulating cysteine rich protein of 12 kDa that belongs to the family of “resistin-like molecules” or “FIZZ” (found in inflammatory zone) [29]. It consists of homodimers connected by disulfide bridges. Resistin is strongly involved in insulin resistance and obesity in rodents but its role in humans is still unclear. In humans, resistin is mainly produced by monocytes and macrophages and less in pancreatic *β*-cells [30] in lung [31], and placental tissue [32], whereas in rodents it is more expressed in adipose tissue [33]. Resistin injection in rodent causes insulin resistance whereas with antibodies against resistin increased the insulin sensitivity in obese mice [28]. These data suggest that resistin induced insulin resistance and an increase in resistin circulating level contributes to decreased insulin sensitivity in obesity. However, in healthy humans, resistin gene expression is very low. Therefore the involvement of resistin in insulin resistance needs to be confirmed. Furthermore, after much research on resistin's mechanism of action its receptor and the signaling pathway involved are still unknown. Recently, Sánchez-Solana et al. suggest that resistin can bind the receptor tyrosine-kinase-like orphan receptor (ROR1) in murine pre 3T3-L1 adipocytes [34]. Benomar et al. suggest also that resistin can bind the Toll-like receptor 4 (TLR4) in the mouse hypothalamus [35]. Thus, in rodents, resistin has opposite effects on the regulation of insulin sensitivity as compared to adiponectin.

### 1.3. Visfatin

Visfatin also known as PBEF (pre-B-cell colony enhancing factor) or Nampt (nicotinamide phosphoribosyltransferase) was discovered in 2005 by Fukuhara et al. [36]. This protein was initially characterized as a growth factor for early-stage B cells [37] and is a 52 kDa protein of 491 amino acids expressed in several tissues including muscle, bone marrow, liver, lymphocytes, and fetal membranes [36] but predominantly in visceral adipose tissue [38]. Fukuhara et al. showed a correlation between visfatin levels in mice and humans and the proportion to the amount of visceral fat [36]. Visfatin presents insulin-mimetic effects, stimulates glucose uptake in adipocytes and muscle cells, and suppresses glucose release from hepatocytes [36, 39]. Visfatin is a rate limiting enzyme involved in NAD biosynthesis from nicotinamide. Like for resistin, visfatin's receptor is still unknown and also the signaling pathway involved. Fukuhara et al. suggested that visfatin can bind to the insulin receptor but at a different site. However this suggestion was later retracted [36].

### 1.4. Chemerin

**Figure 2:**
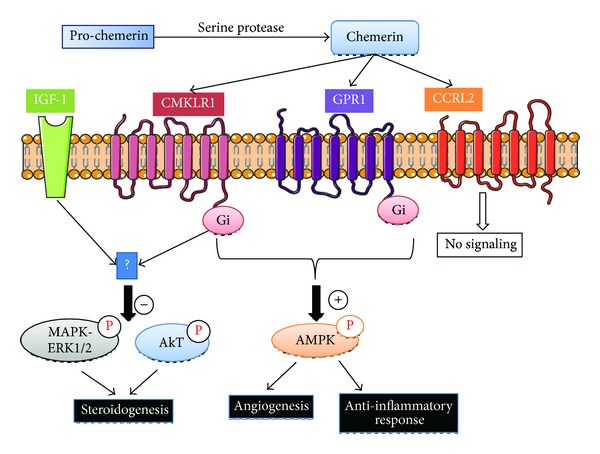
Chemerin receptors, CMKLR1, GPR1, and CCRL2 signaling pathways. Chemerin is able to bind three different G protein-coupled receptors: CMKLR1 (chemokine-like receptor 1), GPR1 (G protein-coupled receptor 1), and CCRL2 (Chemokine (CC motif) receptor-like 2). This latter receptor does not appear to be a signaling receptor. Once activated, CMKLR1 and GPR1 stimulate or inhibit different signaling pathways including MAPK ERK1/2, Akt, and AMPK to regulate different biological processes such as angiogenesis, inflammation, and steroidogenesis. In particular, our group showed that chemerin decreases IGF-1-induced steroid production through MAPK ERK1/2 phosphorylation in human granulosa cells (−: inhibition, +: stimulation).

Chemerin or RARRES2 (retinoic acid responder protein 2) or TIG2 (tazarotene induced gene 2 protein) is a new adipokine of 163 amino acids and a molecular weight of 14 kDa [40]. Chemerin is secreted as a precursor named prochemerin that is cleaved at the C-terminus by a serine protease to become active [40]. Chemerin can bind three G-coupled receptors: CMKLR1, GPR1, and CCRL2 (Figure 2). CMKLR1 (chemokine-like receptor 1) is predominantly expressed by plasmacytoid dendritic cells, monocytes/macrophages, and natural killer [41] whereas GPR1 is mainly expressed in the liver, intestine, kidney, and adipose tissue [42]. CCRL2 is another GPCR ((chemokine (C-C motif)) that presents high expression in lung endothelial cells and less in liver endothelium [43, 44] but it does not induce chemerin signaling [45]. It is suggested that CCRL2 can regulate the bioavailability of chemerin to other chemerin receptors [44].

## 2. Adipokines and Ovary (Figure 3)

### 2.1. Adiponectin

In the ovary, adiponectin has been identified in follicular fluid (woman and sow [46, 47]) and is expressed in different compartments such as the oocyte (rat [48]), the corpus luteum (rat [48]), and the theca cells (chicken [49] and rat [48]). Interestingly, adiponectin was almost undetectable in rat, chicken, and human granulosa cells [46, 48, 49]. AdipoR1 and AdipoR2 receptors have been identified in different cell types of the follicle (oocyte cumulus, granulosa cells, and theca cells) in different species (rat, cow, pig, fish, and chicken) including women [46–51]. However their expression level differs; mRNA AdipoR1 level was higher than that of AdipoR2 in granulosa and theca cells from large follicles, whereas an opposite expression pattern was observed in oocytes from large bovine follicles [52]. In chicken ovarian adiponectin, AdipoR1 and AdipoR2 were expressed in whole ovary [48]. In this species adiponectin mRNA expression was twofold lower in F4 theca cells than in F1 theca cells and opposite results were obtained in granulosa cells. And the expression of adiponectin in theca cells was 10- to 30-fold higher than in granulosa cells [48]. These results show that the expression of adiponectin and its receptors is differentially modulated with cell type and cell maturity.

The role of adiponectin has been studied *in vitro* on steroidogenesis of granulosa and theca cells and oocyte maturation in several species. In primary rat and human granulosa cells, adiponectin increases progesterone and estradiol secretions in response to IGF-1 (insulin-like growth factor 1) [46]. In rats, this increase is due to an activation of IGF-1 receptor signaling and an increase in the protein expression of aromatase [49]. In the human granulosa cell line, KGN, the specific AdipoR1 and AdipoR2 inactivations showed that AdipoR1 is involved in cell survival whereas AdipoR2 is preferentially involved in steroidogenesis [53]. In 2008, Gutman et al. have shown an increase in the concentration of adiponectin in follicular fluid in response to gonadotropin in human ovarian after treatment with recombinant LH (luteinizing hormone) [54]. In cultured bovine theca cells, adiponectin suppresses *in vitro* androstenedione production and gene expression of the LH receptor and key enzymes in the androgen synthesis pathway (CYP11A1 (cytochrome P450, family 11, subfamily A, polypeptide 1) and CYP17A1 (cytochrome P450, family 17, subfamily A, polypeptide 1)) [55, 56]. Moreover in these cells, knockdown of genes for AdipoR1 and AdipoR2 was associated with increased androstenedione secretion [55]. In pigs, adiponectin increases *in vitro* granulosa cell expression of molecules involved in ovulation (COX2 (cyclooxygenase 2), PGE2 (prostaglandin E2), and EGF (epidermal growth factor)) and improves *in vitro* embryo development [47]. During IVF protocol in women and mice, adiponectin improves oocyte maturation and embryo development [57]. Similar results were observed for the *in vitro* development of embryos in pig [58]. In this species, several polymorphisms of adiponectin and its receptors (AdipoR1 and AdipoR2) have been identified. Certain variants and haplotypes identified are associated with larger litters, a smaller number of stillborn and mummified piglets, and shorter weaning-estrus intervals [59].

### 2.2. Resistin

Resistin is expressed in bovine, rat, and human ovarian cells [60–62]. Moreover, resistin modulates granulosa cell function, such as steroidogenesis and proliferation, in basal state or in response to IGF-I *in vitro *[60]. Furthermore, Spicer et al. showed that resistin inhibits steroidogenesis of undifferentiated (small follicles) granulosa cells and inhibits mitogenesis of differentiated (large follicles) granulosa cells collected from cattle [63]. In human cultured theca cells, recombinant resistin triggered 17*α*-hydroxylase activity, a marker of ovarian hyperandrogenism in women with PCOS [64]. Furthermore, recently in pig, resistin increased progesterone, androstenedione, and testosterone secretion by upregulating the steady state levels of CYP11A1, 3betaHSD, CYP17A1, and 17beta HSD. In the latter study, recombinant resistin had no effects on oestradiol secretion and CYP19A expression in ovarian follicles [65]. All these data suggest that resistin could affect *in vivo* ovarian function.

### 2.3. Visfatin

The presence of visfatin in human ovarian follicles has been shown in oocytes, cumulus cells, granulosa, and theca cells [66]. In primary human granulosa cells and KGN cells, visfatin expression (mRNA and protein) is regulated by metformin, an antidiabetic agent through AMPK activation and SIRT1 activity [66]. Furthermore, recombinant human visfatin increases IGF-1-induced steroid secretion and cell proliferation through NAMPT activity in primary human granulosa and KGN cells [66]. Shen et al. have also showed that visfatin expression in primary human granulosa cells is increased by hCG and prostaglandin E2 treatments [67]. In humans, the precise reproductive role of resistin remains controversial. Indeed, Seow et al. showed that resistin was not a major determining factor in the growth and maturation of oocytes during ovarian stimulation [68], whereas Chen et al. (2007) demonstrated a negative correlation between serum resistin levels and the number of oocytes retrieved during IVF [69]. In rodents, a recent study showed that administration of visfatin during superovulation improves the developmental competency of oocytes and fertility potential in old female mice suggesting a role of this adipokine in ovarian function and oocyte quality in older mammals [70].

### 2.4. Chemerin

Chemerin and CMKLR1 are expressed in the mouse, bovine, and human ovary [71, 72]. In humans, our group showed that chemerin and CMKLR1 are present in granulosa and theca cells and follicular fluid [72]. Chemerin levels are significantly higher in follicular fluid than in plasma [72]. *In vitro*, we have shown that rhChem inhibited IGF-1-induced progesterone, E2 secretion, and cell proliferation in human granulosa cells and this was associated with a reduction in the levels of p450 aromatase and a decrease in the tyrosine phosphorylation of IGF-1R*β* subunit and phosphorylation of Akt and MAPK ERK1/2 [72]. In rodents, ovarian and circulating chemerin levels are elevated in a chronically androgenised rat model and chemerin suppresses FSH-induced steroidogenesis [73] and induces apoptosis in granulosa cells, thereby suppressing follicle growth [74]. Furthermore in rat, chemerin suppresses the expression of the oocyte-derived factor, GDF9, that promotes granulosa cell proliferation and preantral/early antral follicle growth. It also suppresses GDF9-induced follicular growth *in vitro*. Thus, chemerin appears as an important intraovarian regulator and could contribute to the dysregulation of follicular development.

## 3. Adipokines in Uterus and Endometrium

In pig, AdipoR1 and AdipoR2 are also highly expressed in the endometrium [12] and both were localized in the endometrial and glandular human epithelium [75]. Interestingly, AdipoR1 and AdipoR2 transcript levels are higher during the midluteal phase suggesting that adiponectin may affect implantation [75]. Adiponectin, AdipoR1, and AdipoR2 are expressed in the uterus in different species (rabbit [76], pig [12], human [75], and rodents ([77]). In human, a strong expression of the receptors was observed in the endometrium during the implantation of the embryo. A recent study indicates that the expression of AdipoR1 and AdipoR2 is decreased (<60%) in the endometrium of women who experience implantation failure compared to fertile women with embryo implantation [78]. In contrast, plasma adiponectin levels are greatly reduced in patients with not only gestational diabetes and polycystic ovary syndrome (PCOS), but also endometriosis and endometrial cancer (cited in [79]). These studies suggest that a change in the expression of adiponectin and/or its receptors may be involved in endometrial receptivity [77, 78]. In addition, deregulation of the expression of this system could occur in certain pathological conditions associated with miscarriage or bad implantation [77]. Chemerin is also expressed and produced by the human uterus [80]. It is differentially expressed by decidual cells during early pregnancy, being present at high levels in stromal cells and extravillous trophoblast cells but not in decidual endothelial cells. Chemerin production in the uterus is upregulated during decidualization suggesting an important role in vascular remodeling during early pregnancy [80]. Visfatin is expressed in pig uterus [81]. However, few data are available to date on the potential role of not only visfatin but also resistin during embryo implantation.

## 4. Adipokines and Placenta

There is evidence indicating that several adipocytokines play an important role in placental function. The expression of adiponectin in the human placenta is contradictory in the literature [82–85]. However, AdipoR1 and AdipoR2 are expressed in placental trophoblasts at the mRNA level [82], but only AdipoR2 protein is reported in human [86] and mouse trophoblast plasma membranes [87]. McDonald and Wolfe demonstrated that globular adiponectin attenuates mRNA expression and/or production of placental lactogen, chorion gonadotropin, and progesterone in trophoblast cells [85]. Adiponectin has been reported to promote syncytialization in BeWo cells and in primary human trophoblastic cells (PHT) isolated from early first trimester placentas [88] but inhibit syncytialization in PHT isolated later in gestation [85, 88]. Adiponectin decreases the *in vitro* proliferation of BeWo and JEG-3 trophoblastic lines, stimulates the differentiation of trophoblast in villous syncytiotrophoblast, and promotes secretion of placental hormones (hCG (human chorionic gonadotropin) and leptin) [89]. Like adiponectin receptors, resistin is present in human placenta especially in trophoblastic cells [32]. In humans, resistin expression increases during the gestation. Indeed, placenta resistin expression is significantly higher at term than in the first trimester [32] and resistin has been reported to play a role in pregnancy. It induces BeWo cell invasiveness and could contribute to the control of placental vascular development [90]. Resistin is also able to modulate glucose transport in human trophoblast cells [91]. Chemerin was detected in human placenta in the third trimester in the cytotrophoblast [92]. It is known that the third trimester of gestation in human is characterized by an anti-inflammatory response, thus chemerin could play a role to induce an anti-inflammatory environment. In the rat, chemerin expression is higher at day 16 and then decreases significantly towards the end of pregnancy [92]. Moreover, in this latter study, rat serum chemerin levels were decreased as gestation progressed [92]. Preeclampsia is a pathology characterized by high blood pressure and significant amounts of protein in the urine of a pregnant woman. As with resistin, maternal chemerin concentrations are significantly higher in preeclampsia patients compared to control patients [93, 94]. In humans, visfatin is also expressed in the placenta [95] where it activates proinflammatory cytokine release and phospholipid metabolism via activation of the NF-*κ*B pathway [84]. In preeclampsia, plasma levels of visfatin and adiponectin do not change even with the severity of the disease [96]. All these data suggest that the adipokines are involved during embryonic implantation and gestation. They could play an important role in the maternal-fetal metabolism and metabolic homeostasis during pregnancy.

## 5. Adipokines: Implications in Infertility Associated with Obesity/Insulin Resistance and Polycystic Ovary Syndrome (PCOS)

The effects of obesity on female reproduction have also been extensively investigated. Several epidemiological studies using large cohorts of pregnant women have demonstrated a link between body mass index and the chances of pregnancy. The risk of taking more than a year to conceive is increased by 27% for overweight women and 78% for obese women [97]. Obese patients, pregnant after infertility treatment, have a significantly higher rate of spontaneous abortion that is correlated to the extent of obesity. Obesity can affect reproductive tract at different levels: oocyte, embryo, placenta, and uterine environment. It is difficult to describe the mechanisms by which obesity or excess weight affects the quality of female gametes. Obesity can affect the quality of gametes by altering the plasma concentrations of reproductive hormones and metabolism. Indeed, the adipose tissue is a site of production of not only steroid hormones but also adipocytokines. Obesity modifies the tissue and/or plasma expression profiles. Obesity is often observed in patients with polycystic ovary syndrome (PCOS) (about 50% of PCOS patients are obese). This syndrome presents both fertility and metabolic disorders. It is one of the most common causes of female infertility, which affects 5–10% of women of reproductive age. It is a heterogeneous syndrome with the characteristics of hirsutism, acne, anovulation, hyperandrogenemia, polycystic ovaries, and infertility [98]. Data regarding the levels of adipokines including adiponectin, resistin, and visfatin in PCOS patients are still controversial (references cited in [10]). Serum adipokines concentrations are reduced in PCOS patients compared with controls in some studies whereas in other reports serum adipokines did not differ between PCOS and control (references cited in [10]). Several meta-analyses have reported the PCOS association with polymorphisms of the adiponectin and resistin genes [99–101]. Although the profile of most adipokines such as adiponectin, resistin, and visfatin is still unknown in PCOS due to the conflicting data, the dysregulated adipokine levels in PCOS patients suggest that adipokines contribute to the pathology of PCOS.

## 6. Concluding Remarks

White adipose tissue can influence and communicate not only with other peripheral organs but also with reproductive tissues through the production of adipokines such as adiponectin, resistin, visfatin, and chemerin. These adipokines are also expressed in the reproductive tract suggesting not only endocrine but also autocrine or paracrine effects of these molecules. It would be important to inhibit each adipokine and/or its receptor in a cell specific manner in order to determine the role of these adipokines in different cells of the reproductive tract. Some adipokines seem to be involved in gestational pathologies like gestational diabetes mellitus and preeclampsia by their impact on insulin sensitivity and energy homeostasis. In our review we focus on female reproductive cells but adipokines and their receptors are also expressed in male gonad and consequently could affect spermatogenesis. The reproductive tract is regulated by hormones produced by the pituitary-hypothalamus axis where some adipokines are also present. Thus, adipokines could influence the central regulation of reproductive function by modulating the LH and FSH secretion. The secretion of adipokines and its influence on PCOS are very controversial. Further investigation is warranted to better understand the relationship between the adipokines and reproductive function.

## Figures and Tables

**Figure 3:**
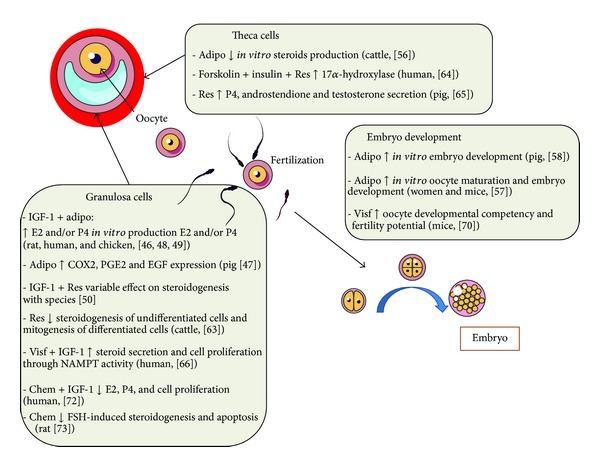
Adiponectin, resistin, visfatin, and chemerin effects on ovarian cells in various species. Adipo: adiponectin, Res: resistin, Visf: visfatin, Chem: chemerin, E2: oestradiol, P4: progesterone, and IGF-1: insulin-like growth factor-1.
